# Dosage consideration for transcranial direct current stimulation in post-stroke dysphagia: A systematic review and network meta-analysis

**DOI:** 10.3389/fneur.2023.1098831

**Published:** 2023-01-24

**Authors:** Jianwei Xie, Chiteng Zhou, Gilbert Ngaruwenayo, Minghui Wu, Xiaoyu Jiang, Xiaohan Li

**Affiliations:** ^1^School of Nursing, China Medical University, Shenyang, China; ^2^The First Clinical College, China Medical University, Shenyang, China; ^3^General Hospital of Pingmei Shenma Group, Pingdingshan, China

**Keywords:** dosage consideration, transcranial direct current stimulation, post-stroke dysphagia, systematic review, network meta-analysis

## Abstract

**Objective:**

This systematic review and network meta-analysis sought to determine the efficacy of different intensities of transcranial direct current stimulation (tDCS) in patients with dysphagia after stroke to improve swallowing function.

**Methods:**

Randomized-controlled trials (RCTs) of tDCS in post-stroke dysphagia were searched from Pubmed, EMBASE, Cochrane Library, Web of Science, China National Knowledge Infrastructure (CNKI), Chinese Biomedical Literature Service System (SinoMed), Wanfang database, and Chinese Scientific Journals Database (VIP) from databases' inception to June 22, 2022. Article screening, data extraction, and article quality evaluation were completed by 2 independent researchers. Network meta-analysis was performed using Stata.

**Results:**

A final total of 20 studies involving 838 stroke patients were included. The included control interventions were sham tDCS and conventional therapy (CT). Network meta-analysis showed that 20 min of 1.2, 1.4, 1.5, 1.6, and 2 mA anodal tDCS and 30 min of 2 mA anodal tDCS significantly improved post-stroke dysphagia compared with CT (all P < 0.05). In addition, 20 min of 1, 1.4, 1.6, and 2 mA anodal tDCS also significantly improved post-stroke dysphagia compared with sham tDCS (all *P* < 0.05). Our results demonstrated that 20 min of stimulation at 1.4 mA was the optimal parameters for anodal tDCS and exhibited superior efficacy to CT [SMD = 1.08, 95% CI (0.46, 1.69)] and sham tDCS [SMD = 1.45, 95% CI (0.54, 2.36)].

**Conclusion:**

Different durations and intensities of anodal tDCS are effective in improving post-stroke dysphagia. However, 20 min of tDCS at 1.4 mA may be the optimal regimen.

**Systematic review registration:**

https://www.crd.york.ac.uk/PROSPERO/#recordDetails, identifier CRD42022342506.

## Introduction

Stroke is a clinical syndrome caused by cerebrovascular disorder and a major disease that seriously threatens human health (1). A recent study showed that there are nearly 113 million stroke-associated disability-adjusted life years worldwide (2). Dysphagia is a common stroke complication (3) found in 20–45% of patients (4, 5). The manifestation of dysphagia differs with varying stroke site, and general cerebral cortex lesions or injuries are common causes of dysphagia (6). Untimely treatment of dysphagia can lead to complications such as aspiration pneumonia, malnutrition, dehydration and suffocation (5, 7), which seriously affects the prevention and treatment of dysphagia and poses a great challenge for medical staff (8, 9). Therefore, how to improve the swallowing functions of patients in the early stage of stroke and their survival remains an actively investigated topic in clinical research.

There are pharmaceutical and non-pharmaceutical treatments for post-stroke dysphagia. Non-pharmaceutical treatments include swallowing exercises, dietary adjustments, feeding exercises, traditional rehabilitation, glacial acid stimulation, balloon dilation, acupuncture, electroacupuncture, neuromuscular electrical stimulation, behavior management, electromyographic biofeedback, and non-invasive brain stimulation (NIBS). In particular, NIBS has been shown to promote better recovery from post-stroke dysphagia than other non-pharmaceutical treatments (10). Transcranial direct current stimulation (tDCS) is a type of NIBS that transcranially delivers a steady flow of low-intensity current (usually within the range of 1–2 mA) through two relatively large electrodes placed on the scalp. Application of a direct current to the projected area on the scalp modulates neuronal resting potential threshold and intervenes with neuron firing. Neuron firing increases when the anode is close to the neuron or dendrite, and decreases when the cathode is close to the neuron or dendrite. Therefore, the therapeutic effect of tDCS is mediated by altering the excitability of the cerebral cortex within the stimulated area through the anode and cathode (11). Due to its non-invasiveness, ease of operation, minimal side effect, ease of equipment management and low cost, tDCS has been widely used in the treatment of various dysfunctions in stroke patients and has demonstrated promising clinical application prospects (12–14). The efficacy of tDCS is highly dependent on the parameters of stimulation, including stimulation intensity, frequency, duration, and electrode placement. Stimulation intensity and duration are the common factors for tDCS and the major factors that influence tDCS safety and efficacy. The optimal stimulation intensity and duration for tDCS are currently unclear. Jefferson et al. (15) conducted neuroelectrophysiological observations on the optimal stimulation parameters of tDCS in the pharyngeal motor cortex and found that 10 min of 1.5 mA and 20 min of 1.0 mA anodal tDCS effectively induced excitation of the pharyngeal motor cortex. Nitsche and Paulus (16) reported that increased stimulation intensity (higher current intensity and longer stimulation time) can lead to better treatment efficacy. However, another study showed that prolonged tDCS stimulation may induce neural adaptation, resulting in decreased neural excitation (17). The guidelines by Bikson et al. (18) recommended a tDCS stimulation intensity of 1–4 mA, but the optimal stimulation intensity and duration were not mentioned. Several published meta-analyses have compared the efficacy of tDCS in improving post-stroke dysphagia (19–21), but there are no evidence-based findings on the optimal current intensity and stimulation time of tDCS in post-stroke dysphagia. Therefore, in order to examine the effect of different stimulation time and current intensities on the efficacy of tDCS in post-stroke dysphagia, we conducted a network meta-analysis of key stimulation parameters (stimulation intensity and duration) of tDCS in existing studies.

In this study, we performed a network meta-analysis of the efficacy of different stimulation time and current intensities of tDCS in post-stroke dysphagia in order to provide reliable support for clinical application.

## Data and methods

This study was conducted in accordance with the PRISMA-network meta-analysis extension (Supplementary material 1) (22) and registered on PROSPERO (CRD42022342506).

### Search strategy

Randomized-controlled trials (RCTs) of tDCS in post-stroke dysphagia were searched in PubMed, Embase, Cochrane Library, Web of Science, CNKI, Wanfang Data, VIP, and CBM from inception to June 22, 2022 by two researchers (Jianwei Xie and Minghui Wu). Each database was searched using medical subject headings (MeSH) and free search terms. The following search terms were employed: (“stroke” OR “cerebrovascular accident” OR “apoplexy” OR “brain vascular accident” OR “cerebral vascular accident” OR “hemiplegia” OR “CVA” OR “thrombotic stroke”) AND (Deglutition Disorders OR “aphagopraxia” OR “deglutition difficulty” OR “dysphagia” OR “difficulty swallowing”) AND (“transcranial direct current stimulation” OR “Transcranial Electrical Stimulations”). The specific search strategy is described in Supplementary material 2.

### Inclusion criteria

(1) Participants: Age > 18 years, definitely diagnosed with dysphagia by CT or MRI and the stroke type was either cerebral hemorrhage or cerebral infarction.
(2) Intervention: Anodal tDCS.
(3) Control treatment: Conventional therapy (CT) or sham tDCS.
(4) Outcomes: The primary outcome measure was the assessment of swallowing function before and after tDCS, and all scales for assessing dysphagia in stroke were included.
(5) Study type: RCTs.

### Exclusion criteria

Any other types of articles, such as systematic reviews, letters, case reports, editorials, animal studies, comments, and non-RCTs; Studies that combined other intervention(s); Studies involving patients with severe aphasia or cognitive impairment; Studies with incomparable baseline data or have not reported baseline data; Studies with poor design or improper statistical analysis; Studies with incomplete data or source data or full-text files could not be obtained after contacting the corresponding author; Studies without corresponding results; Studies with unclear diagnostic criteria and intervention time.

### Data extraction

Article screening and data extraction were completed by two independent researchers (Chiteng Zhou and Xiaoyu Jiang) according to the study objective and inclusion criteria and cross-verified. In the case of any disagreement, a third researcher (Minghui Wu) was consulted. Data were extracted using a customized data extraction form and included (1) General information of included studies; (2) Baseline characteristics of subjects; (3) Details of intervention; (4) Outcome measures and assessment tools; (5) Methodological quality of the included studies. Missing information were requested from the corresponding author through email or telephone.

### Risk of bias of included studies

The risk of bias of the included RCTs was assessed by two researchers (Jianwei Xie and Gilbert Ngaruwenayo) using the Cochrane Handbook for Systematic Review (version 5.1.0) (23). The items of assessment included random sequence generation, allocation concealment, blinding of participants and personnel, blinding of outcome assessment, incomplete outcome data, blinding of outcome assessment personnel, selective reporting of study results, and other bias. Each item was evaluated as “low risk of bias,” “unclear risk of bias,” and “high risk of bias.” If all of the above criteria are met, the risk of bias is minimal. If the above criteria are partially met, the risk of bias is moderate. If none of the above criteria are met, the risk of bias is high. A risk of bias graph was generated using Rev Man 5.4.

### Statistical analysis

Data were analyzed using Stata 15.0 (StataCorp LLC, College Station, Texas). First, meta-analysis was performed to directly compare different interventions. Since outcome measures are continuous variables and there are differences among questionnaires, standardized mean difference (SMD) and 95% confidence interval (CI) were used for analyses. If the 95% CI does not include 0, a *P* < 0.05 is considered statistically significant. Heterogeneity among studies was assessed by *Q*-test and *I*^2^. Heterogeneity is not significant when *P* > 0.05 and *I*^2^ < 50%, and a fixed effects model is used for meta-analysis. When *P* < 0.05 or *I*^2^ > 50%, the source of heterogeneity was analyzed. Once clinically significant heterogeneity was excluded, a random effects model was used to combine the data. Sensitivity analysis was conducted by sequentially removing each included study. If pairwise comparison does not lead to significant change in the meta-analysis results, this indicates that the results are relatively stable. Articles that have a relatively large impact on the study results were excluded by sensitivity analysis.

Random effects network meta-analysis was conducted using the Frequentist approach. This approach uses the inverse variance to combine direct and indirect evidence, that is, the inverse variance of each study is used as the weight and the weighted mean is calculated for the effect of each study. The variance of the overall effect is the reciprocal of the sum of weights (24). A network diagram provides an intuitive comparison of different interventions. The size of each dot represents the total sample size of an intervention, and the thickness of the line between two dots represents the number of studies that directly compared the two interventions. The absence of a connecting line indicates no direct comparative study. Consistency in network meta-analysis refers to the similarity between the results of direct and indirect comparisons. When there is a closed loop in the evidence network, consistency is evaluated using the node-splitting method. A *P* > 0.05 indicates non-significant inconsistency between direct and indirect comparisons (25). Different intensities of the intervention were compared in a pairwise manner, and a *P* < 0.05 was considered to be statistically significant. The efficacy of each intervention was ranked by surface under the cumulative ranking (SUCRA). SUCRA can range from 0 to 100%, and a greater SUCRA indicates better efficacy. Publication bias of the included articles was evaluated using a funnel plot and Egger's test. Asymmetry in the funnel plot and *P* < 0.05 in the Egger's test indicate publication bias in the included articles (26).

## Results

### Search results

**Figure 1:**
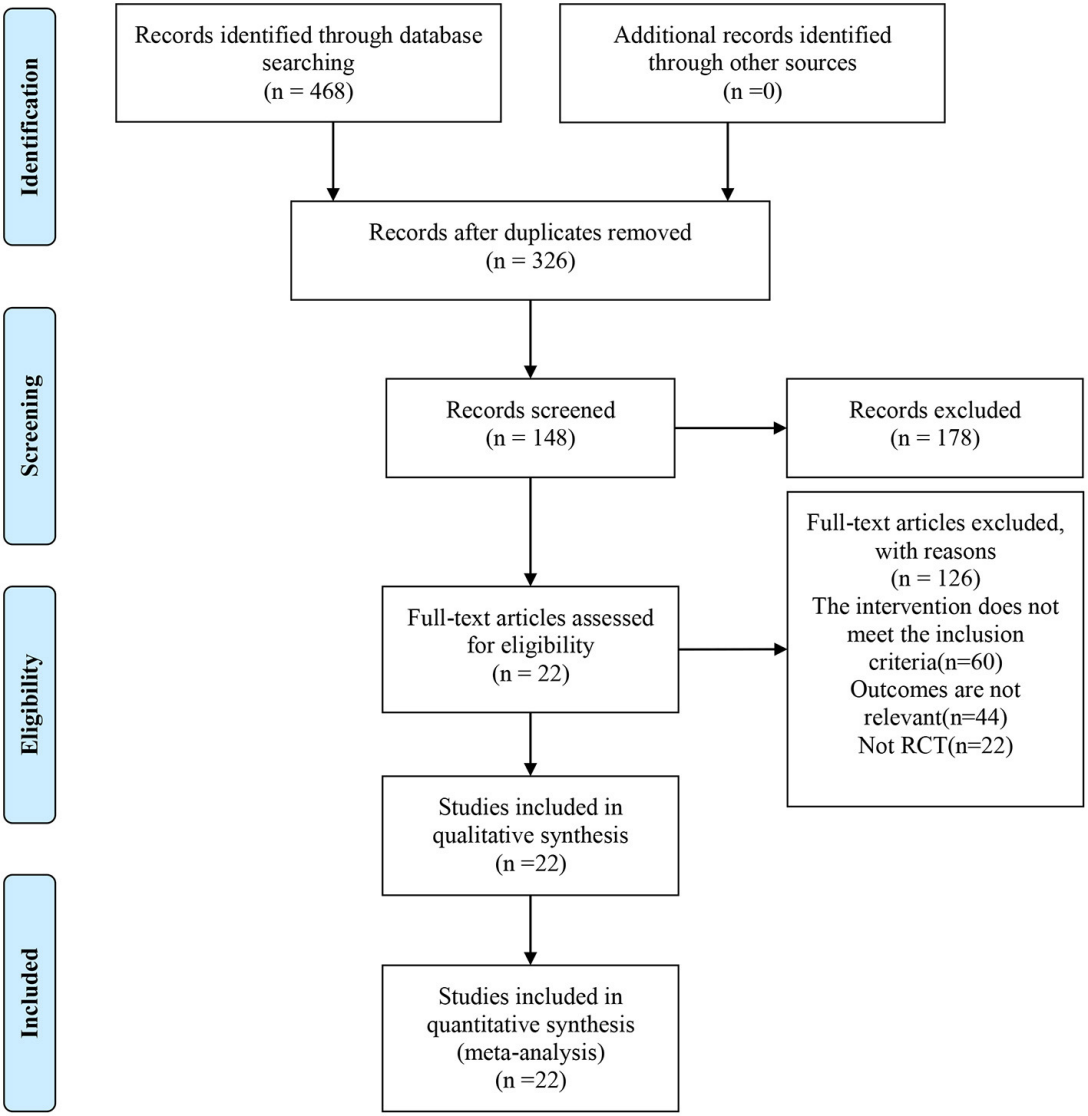
Flow diagram for article screening.

A total of 468 relevant articles were identified through initial search, 326 articles were obtained after removal of duplications by Endnote, and 148 articles remained after reading the study title and excluding studies that did not meet the criteria for study design, subject and outcome measures. A final total of 22 RCTs were included after full-text reading and further screening (27–48). The flow diagram for study inclusion is shown in Figure 1.

### General characteristics of included studies

Of the 22 included RCTs, 16 compared the efficacy of anodal tDCS and sham tDCS, and 6 compared the efficacy of anodal tDCS and CT. The included 22 RCTs were published between 2013 and 2022 and involved 928 stroke patients. The current intensity of tDCS used included 1, 1.2, 1.4, 1.5, 1.6, and 2 mA; stimulation durations were 20 and 30 min; and the shortest and longest treatment duration were 5 days and 2 months, respectively. For assessment of dysphagia, 6 RCTs used the Dysphagia Outcome and Severity Scale (DOSS), 8 RCTs used the modified Mann Assessment of Swallowing Ability (MMASA), 4 RCTs used the Functional Oral Intake Scale, 2 RCTs used the Videofluoroscopic Swallowing Study (VFSS), 1 RCT used the Functional Dysphagia Scale (DFS), and 1 RCT used the Wada Drinking Water Test (WTDWT). Detailed characteristics of all included studies are summarized in Table 1.

**Table 1:** General characteristics of the included studies.

**Study**	**Country**	**Sample**		**Age (years)**		**Gender (M/F)**		**Stroke onset**		**Intervention**		**tDCS protocol**				**Stroke type**	**Stroke location**	**Primary outcome**
		**Control group**	**tDCS group**	**Control group**	**TDCS GROUP**	**Control group**	**tDCS group**	**Control group**	**tDCS group**	**Control group**	**tDCS group**	**Intensity of stimulation **	**Duration of stimulation **	**Treatment period**	**Site of stimulation**			
Kumar et al. (28)	United State	7	7	70 ± 11.96	79.71 ± 10.21	4/3	3/4	(96.71 ± 45.93) h	(80.29 ± 42.31) h	Sham tDCS	Anodal tDCS	2 mA	30 min	Once a day for 5 days	Undamaged hemisphere	CI	Unilateral hemisphere	DOSS
Ahn et al. (29)	Korea	13	13	66.38 ± 10.67	61.62 ± 10.28	6/7	9/4	(11.62 ± 4.56) m	(12.27 ± 4.92) m	Sham tDCS	Anodal tDCS	1 mA	20 min	Five times per week for 2 weeks	Bilateral pharyngeal motor cortex	CI	Unilateral cortical or subcortical	DOSS
Mao et al. (30)	China	20	20	61.25 ± 8.02	59.80 ± 7.27	44,785	11/9	(3.60 ± 2.49) m	(3.25 ± 2.24) m	CT	Anodal tDCS	1.6 mA	20 min	Six times per week for 8 weeks	Undamaged pharyngeal motor cortex	CH + CI	Brain stem	DOSS
Shigematsu et al. (31)	Japan	10	10	64.7 ± 8.9	66.9 ± 6.3	7/3	7/3	At least 1 month	NA	Sham tDCS	Anodal tDCS	1 mA	20 min	10 days	Affected pharyngeal motor cortex	CH + CI	Unclear	DOSS
Pingue et al. (40)	Italy	20	20	67.78 ± 8.78	64.49 ± 16.56	8/12	12/8	NA	NA	Sham tDCS	Anodal tDCS	2 mA	30 min	NA	Affected pharyngeal motor cortex	CH + CI	Unclear	DOSS
Sawan et al. (41)	Egypt	20	20	50.30 ± 5.22	53.30 ± 5.03	NA	NA	NA	NA	Sham tDCS	Anodal tDCS	2 mA	30 min	Five consecutive sessions for 2 weeks	Bilateral pharyngeal motor cortex	CH	Unilateral hemisphere	DOSS
Chen et al. (42)	China	41	43	56.32 ± 7.81	54.31 ± 7.80	23/18	25/18	3.21 ± 0.67	3.22 ± 0.58	CT	Anodal tDCS	1.2 mA	20 min	Five times per week for 2 weeks	Affected oropharyngeal cortex	CH + CI	Unilateral hemisphere	MMASA
Chen et al. (43)	China	14	14	63 ± 12.1	65 ± 9.5	12/2	13/1	(98 ± 9.1)d	(102 ± 15.5)d	Sham tDCS	Anodal tDCS	1 mA	20 min	Five times per week for 4 weeks	Left mastoid region	CH + CI	Unclear	MMASA
Tong et al. (44)	China	31	31	62.80 ± 7.47	63.03 ± 7.69	22/9	24/7	(59.45 ± 13.91)d	(56.93 ± 16.63)d	Sham tDCS	Anodal tDCS	1 mA	20 min	Five times per week for 2 weeks	Affected pharyngeal motor cortex	CH + CI	Unclear	MMASA
He et al. (47)	China	8	7	60.25 ± 18.59	61.86 ± 8.13	6/2	6/1	(1.92 ± 0.24) m	(1.8 ± 0.6) m	Sham tDCS	Anodal tDCS	1.4 mA	20 min	Once a day for 10 days	Bilateral cerebellar hemisphere	CH + CI	Unilateral hemisphere	MMASA
Yuan et al. (48)	China	15	15	57.4 ± 7.2	60.7 ± 11.5	13/2	14/1	(58.5 ± 28.5) d	(57.7 ± 25.8) d	Sham tDCS	Anodal tDCS	1 mA	20 min	Once a day for 20 days	Bilateral cerebellar hemisphere	CH + CI	Cerebellum	MMASA
Wang et al. (27)	China	20	20	60.8 ± 11.2	64.8 ± 7.2	15/5	13/7	(47.9 ± 21.6) d	(51.2 + 28.9) d	Sham tDCS	Anodal tDCS	1.5 mA	20 min	Five times per week for 2 weeks	Undamaged pharyngeal motor cortex	CH + CI	Basal ganglia	MMASA
Chen et al. (35)	China	30	30	62.93 ± 4.12	61.27 ± 4.52	19/11	17/13	(1.92 ± 0.24) m	(1.89 ± 0.17) m	CT	Anodal tDCS	1.4 mA	20 min	Five times per week for 2 weeks	Bilateral pharyngeal sensory-motor cortex	CH + CI	Unclear	MMASA
Hua et al. (36)	China	40	40	61.28 ± 10.15	60.29 ± 9.48	29/11	31/9	(48.16 ± 9.97) d	(47.39 ± 10.83) d	CT	Anodal tDCS	1 mA	20 min	Ten times per week for 4 weeks	Bilateral pharyngeal motor cortex	CH + CI	Basal ganglia	MMASA
Suntrup-Krueger et al. (32)	Germany	30	29	67.2 ± 14.5	68.9 ± 11.5	17/13	17/12	(116.8 ± 64.9) h	(116.3 ± 98.9) h	Sham tDCS	Anodal tDCS	1 mA	20 min	Once a day for 4 days	Affected pharyngeal motor cortex	CI	Supratentorial; infratentorial	FOIS
Wang et al. (33)	China	14	14	62.00 ± 10.46	61.43 ± 11.24	10/4	11/3	(67.50 ± 47.62)d	(66.79 ± 38.6)d	Sham tDCS	Anodal tDCS	1 mA	20 min	Five times per week for 4 weeks	Bilateral esophageal coritical area	CH + CI	Brainstem	FOIS
Kumar et al. (38)	United State	15	14	73 ± 14.1	68 ± 12.6	6/9	3/11	(84.9 ± 39.6) h	(78 ± 33.1) h	Sham tDCS	Anodal tDCS	2 mA	20 min	Twice daily over 5 consecutive days	Unaffected pharyngeal motor cortex	CH	Unclear	FOIS
Farpour et al. (39)	Iran	22	22	70.68 ± 16.33	65.32 ± 16.34	10/12	13/9	(4.50 ± 3.96)d	(4.09 ± 3.97)d	Sham tDCS	Anodal tDCS	2 mA	20 min	One session in a day for 5 day	Contralateral supraorbital region	CH	Supratentorial	FOIS
Li et al. (37)	China	24	23	63.38 ± 8.41	62.87 ± 8.71	15/9	15/8	(37.60 ± 36.84)d	(42.51 ± 61.63)d	Sham tDCS	Anodal tDCS	1.4 mA	20 min	Twice daily for 15 days	Bilaterally oropharyngeal sensory-motor cortex	CH + CI	Brain stem	VFSS
Liu et al. (46)	China	25	25	54.92 ± 3.82	55.82 ± 3.74	15/10	14/11	(14~90) d	NA	CT	Anodal tDCS	1.2 mA	20 min	Five times per week for 2 weeks	Damaged pharyngeal cortex	CH + CI	Unclear	VFSS
Yang et al. (34)	Korea	7	9	70.57 ± 8.46	70.44 ± 12.59	3/4	6/3	(26.9 ± 7.8)d	(25.2 ± 11.5)d	Sham tDCS	Anodal tDCS	1 mA	20 min	five Times per week for 2 weeks	Affected pharyngeal motor cortex	CH	Unclear	FDS
Zhang et al. (45)	China	38	38	62.84 ± 8.02	63.18 ± 7.47	21/17	23/15	(4.19 ± 1.23)w	(4.18 ± 1.21)w	CT	Anodal tDCS	1 mA	20 min	Once a day for 2 weeks	Mastoid region	CH	Unclear	WTDWT

d, day; h, hour; m, month; CI, cerebral infarction; CH, cerebral hemorrhage; CT, conventional therapy; tDCS, transcranial direct current stimulation; DOSS, dysphagia outcome and severity scale; MMASA, modified Mann assessment of swallowing ability; FOIS, functional oral intake scale; VFSS, videofluoroscopic swallowing study; FDS, functional dysphagia scale; WTDWT, Wada drinking water test.

### Methodological quality evaluation

**Figure 2:**
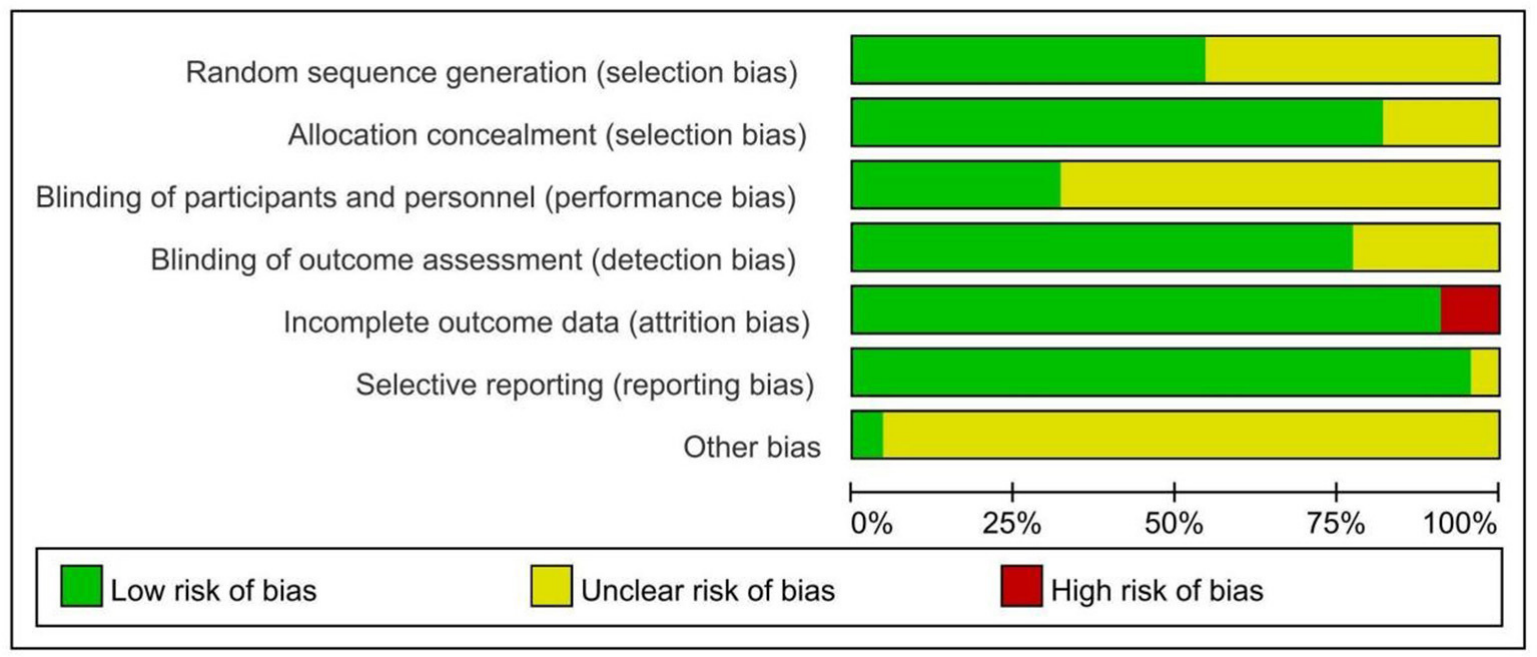
Quality assessment of the risk of bias of included studies.

Among the 22 included RCTs, 12 reported the specific method for random sequence generation (primarily using the random number table), 18 reported allocation concealment, 8 reported blinding of participants and personnel, and 16 reported blinding of outcome assessment. One RCT did not show selective reporting and 2 RCTs did not describe subjects who were lost to follow-up. In addition, other bias was unclear for all studies (Figure 2).

### Direct meta-analysis

There were nine interventions included, namely CT, sham tDCS, 20 min of anodal tDCS at 6 different current intensities (1, 1.2, 1.4, 1.5, 1.6, and 2 mA), and 30 min of 2 mA anodal tDCS (different intensities and durations of tDCS will hereafter be referred to as 20 min + 1 mA anodal, 20 min + 1 mA anodal, 30 min + 2 mA anodal and so on).

**Figure 3:**
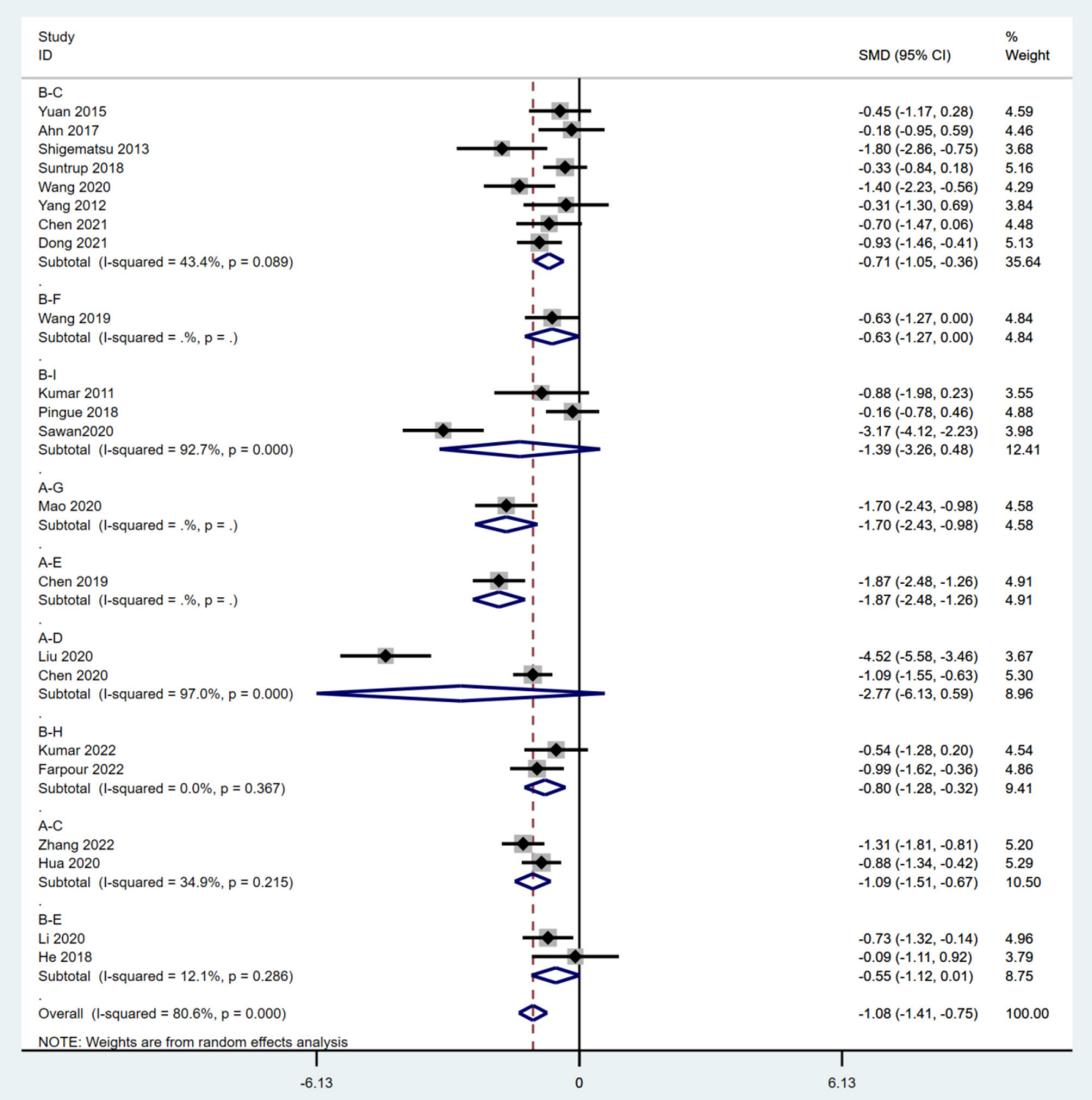
Meta-analysis of pairwise comparisons. A, CT; B, sham; C, 20 min + 1 mA anodal; D, 20 min + 1.2 mA anodal; E, 20 min + 1.4 mAanodal; F, 20 min + 1.5 mA anodal; G, 20 min + 1.6 mA anodal; H, 20 min + 1.6 mA anodal; I, 30 min + 2 mA anodal; SMD, standardized mean difference; CI, confidence interval.

Meta-analysis of direct comparisons showed that 20 min + 1 mA anodal and 20 min + 1.4 mA anodal resulted in significantly better recovery of swallowing functions than sham [SMD = −0.71; 95% CI (−1.05, −0.36) vs. SMD = −0.55; 95% CI (−1.12, −0.01)]. Twenty minutes + 1 mA anodal also led to significantly better recovery of swallowing than CT [SMD = −1.09; 95% CI (−1.51, −0.67)] (Figure 3). Sensitivity analysis of each study revealed that Sawan 2020 and Liu 2020 had the greatest impact on the study results, and hence these two studies were excluded to ensure result stability. A final total of 20 RCTs (27–36, 38–40, 42–48) were included in the network meta-analysis.

### Network meta-analysis

#### Network results

**Figure 4:**
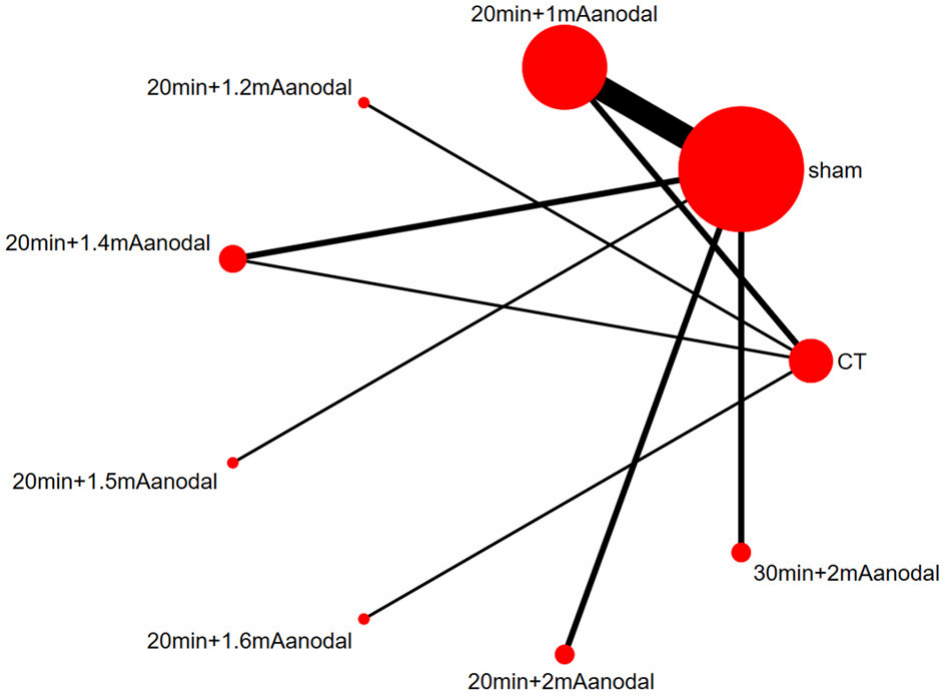
Network diagram.

A network diagram was generated based on the results of each study (Figure 4). A line between two dots indicates direct comparison of the two interventions, and the absence of a line indicates no direct comparison. The thickness of the line indicates the number of studies that directly compared the two interventions, and the size of the dot indicates the sample size of the study. The network diagram showed large sample sizes for sham and 20 min+1 mA anodal, and there were numerous studies that compared the two interventions. In addition, there was also a closed loop.

#### Consistency test

**Figure 5:**
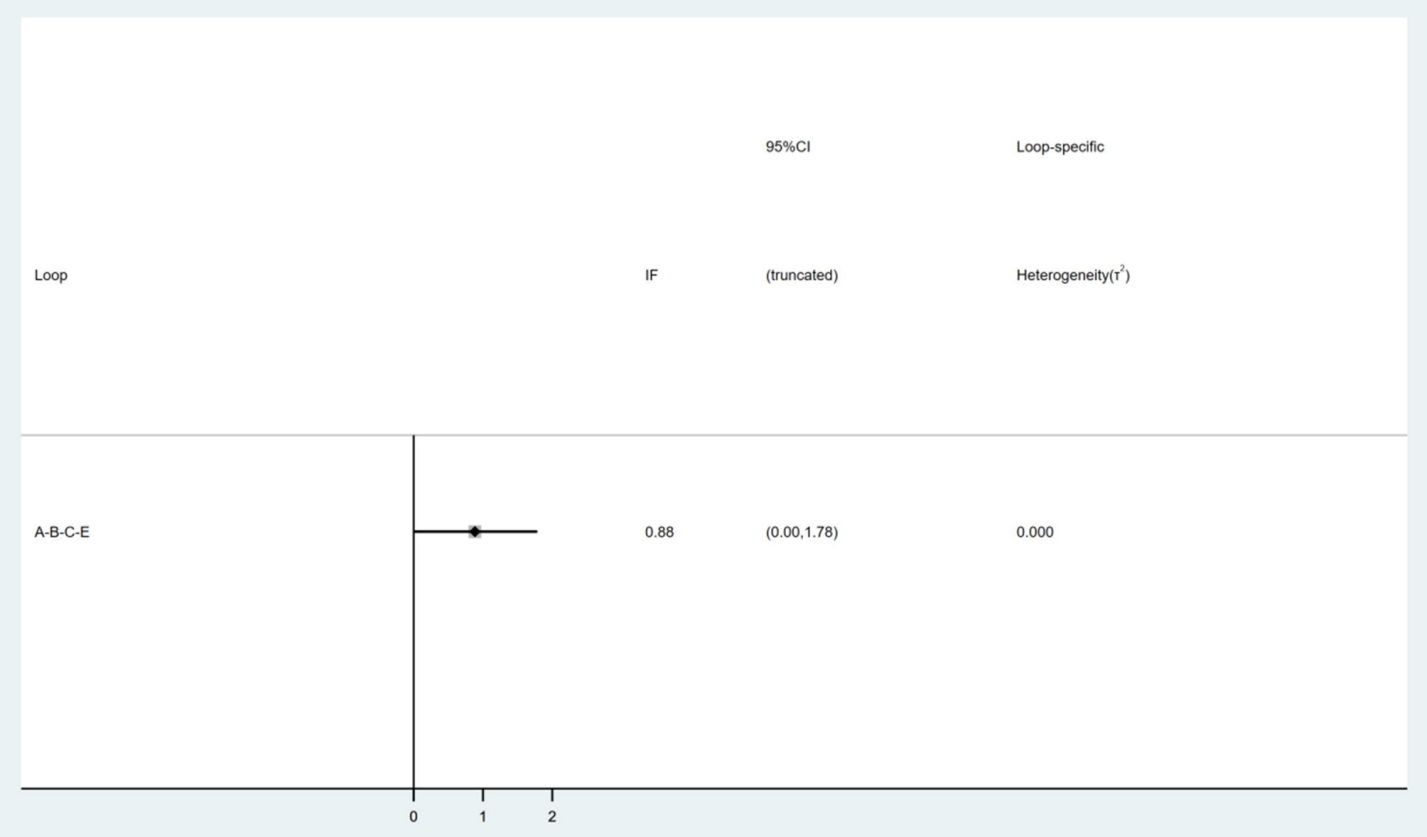
Consistency test. A, CT; B, sham; C, 2 min + 1 mA anodal; E, 20 min + 1.2 mA anodal.

In the inconsistency test, a *P* = 0.1 and > 0.05 indicates consistency. All *P* > 0.05 in the partial inconsistency test. Nine interventions formed a triangle loop (Figure 5). Consistency test showed that the IF of the loop was 0.88 and the 95% CI of the IF included the neutral value (0), which indicates that there was no inconsistency within the triangle loop.

#### Network meta-analysis of different interventions

The prediction intervals for the network meta-analysis of different interventions are shown in Table 2. Twenty minutes + 1.4 mA anodal [SMD = 1.08, 95% CI (0.46, 1.69)], 20 min + 16 mA anodal [SMD = 1.02, 95% CI (0.10, 1.96)], 20 min + 2 mA anodal [SMD = 1.67, 95% CI (0.83, 2.51)], 20 min + 1 mA anodal [SMD = 0.67, 95% CI (0.38, 0.97)], 20 min + 1.2 mA anodal [SMD = 1.08, 95% CI (0.63, 1.52)], 20 min + 1.5 mA anodal [SMD = 1.85, 95% CI (1.12, 2.58)], and 30 min + 2 mA anodal [SMD = 1.18, 95% CI (0.40, 1.95)] demonstrated superior efficacy in swallowing functions than CT. Furthermore, 20 min + 1.4 mA anodal [SMD = 1.45, 95% CI (0.54, 2.36)], 20 min + 1.6 mA anodal [SMD = 1.27, 95% CI (0.28, 2.26)], 20 min + 2 mA anodal [SMD = 0.77, 95% CI (0.21, 1.34)], and 20 min + 1 mA anodal [SMD = 0.67, 95% CI (0.38, 0.97)] resulted in better swallowing outcome than sham tDCS. However, there was no significant difference in efficacy among other interventions (*P* > 0.05).

**Table 2:** Network meta-analysis of various interventions.

**20 min + 1.4 m Aanodal**	**−0.18 (−1.29, 0.93)**	**−0.67(−1.74, 0.39)**	**−0.77(−1.63, 0.08)**	**−0.77 (−1.73, 0.18)**	**−0.83 (−2.01, 0.35)**	**−1.11(−2.21, −0.0)**	**−1.45 (−2.36, −0.54)**	**−1.08 (−1.69, −0.46)**
0.18 (−0.93, 1.29)	**20 min** **+** **1.6 m Aanodal**	−0.49(−1.63, 0.65)	−0.59(−1.54, 0.35)	−0.59 (−1.63, 0.44)	−0.65 (−1.89, 0.60)	−0.93(−2.10, 0.25)	−1.27 (−2.26, −0.28)	−1.02 (−1.95, −0.10)
0.67 (−0.39, 1.74)	0.49 (−0.65, 1.63)	**20 min** **+** **2 m Aanodal**	−0.10(−0.74, 0.54)	−0.10 (−1.09, 0.89)	−0.15 (−1.09, 0.79)	−0.43(−1.28, 0.42)	−0.77 (−1.34, −0.21)	−1.67 (−2.51, −0.83)
0.77 (−0.08, 1.63)	0.59 (−0.35, 1.54)	0.10 (−0.54, 0.74)	**20 min** **+** **1 m Aanodal**	0.00 (−0.76, 0.76)	−0.05 (−0.87, 0.76)	−0.33(−1.03, 0.36)	−0.67 (−0.97, −0.38)	−0.40 (−0.93, 0.13)
0.77 (−0.18, 1.73)	0.59 (−0.44, 1.63)	0.10 (−0.89, 1.09)	−0.00(−0.76, 0.76)	**20 min** **+** **1.2 m Aanodal**	−0.05 (−1.16, 1.06)	−0.33(−1.36, 0.69)	−0.68 (−1.49, 0.14)	−1.08 (−1.52, −0.63)
0.83 (−0.35, 2.01)	0.65 (−0.60, 1.89)	0.15 (−0.79, 1.09)	0.05 (−0.76, 0.87)	0.05 (−1.06, 1.16)	**20 min** **+** **1.5 m Aanodal**	−0.28(−1.27, 0.71)	−0.62 (−1.38, 0.13)	−1.85 (−2.58, −1.12)
1.11 (0.00, 2.21)	0.93 (−0.25, 2.10)	0.43 (−0.42, 1.28)	0.33 (−0.36, 1.03)	0.33 (−0.69, 1.36)	0.28 (−0.71, 1.27)	**30 min** **+** **2 m Aanodal**	−0.34 (−0.98, 0.29)	−1.18 (−1.95, −0.40)
**1.45 (0.54, 2.36)**	**1.27 (0.28, 2.26)**	**0.77 (0.21, 1.34)**	**0.67 (0.38, 0.97)**	0.68 (−0.14, 1.49)	0.62 (−0.13, 1.38)	0.34 (−0.29, 0.98)	**Sham**	−0.92 (−2.01, 0.17)
**1.08 (0.46, 1.69)**	**1.02 (0.10, 1.95)**	**1.67 (0.83, 2.51)**	**0.40 (−0.13, 0.93)**	**1.08 (0.63, 1.52)**	**1.85 (1.12, 2.58)**	**1.18 (0.40, 1.95)**	0.92 (−0.17, 2.01)	**CT**

The numbers in bold are statistically significant.

#### Ranking of intervention efficacy

**Figure 6:**
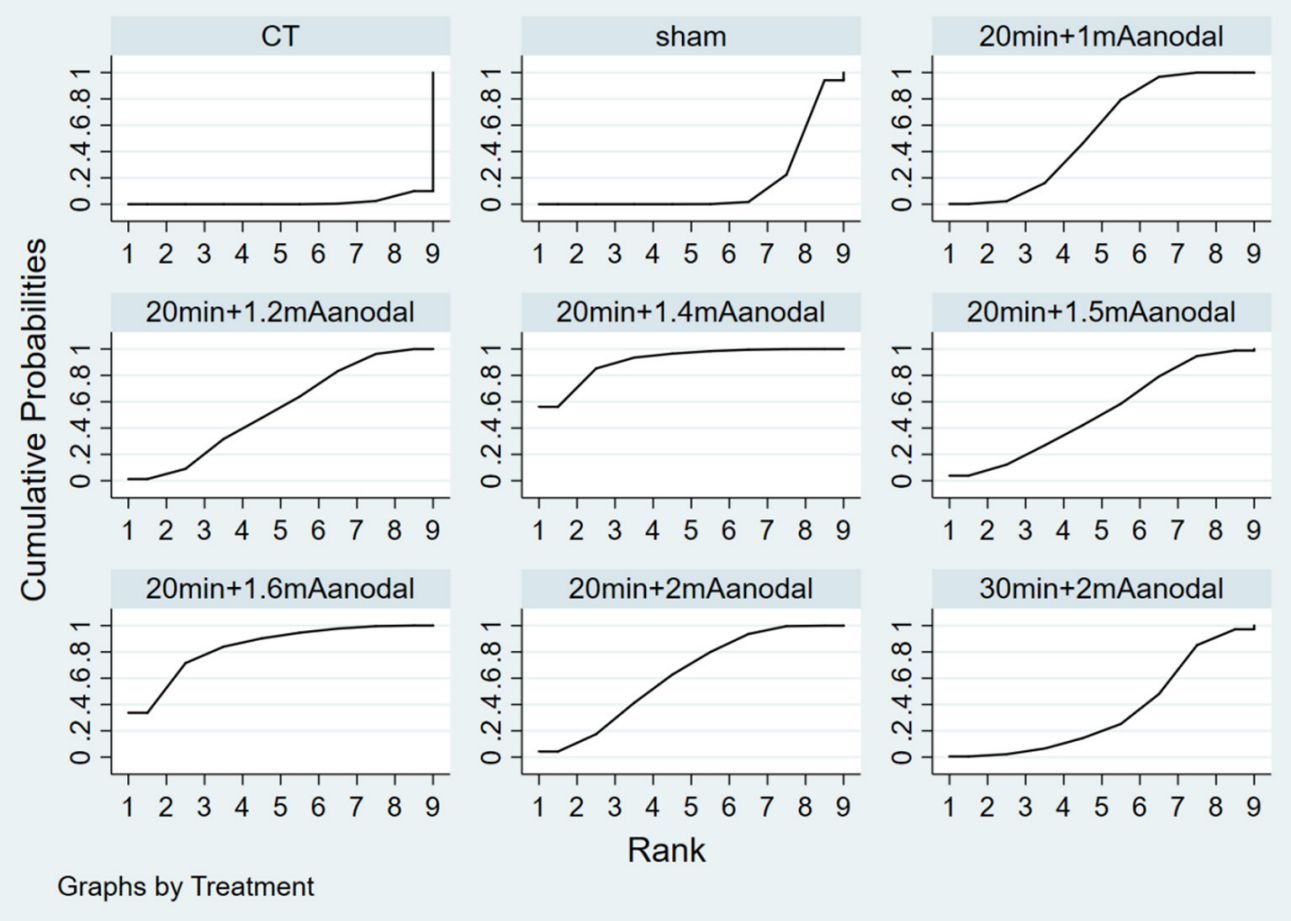
SUCRA rank diagram.

Figure 6 depicts the probability ranking of all treatment protocols. The most likely ranking of the best treatment to the least effective treatment is: 20 min + 1.4 mA anodal > 20 min + 1.6 mA anodal > 20 min + 2 mA anodal > 20 min + 1 mA anodal > 20 min + 1.2 mA anodal > 20 min + 1.5 mA anodal > 30 min + 2 mA anodal > sham > CT.

#### Publication bias

**Figure 7:**
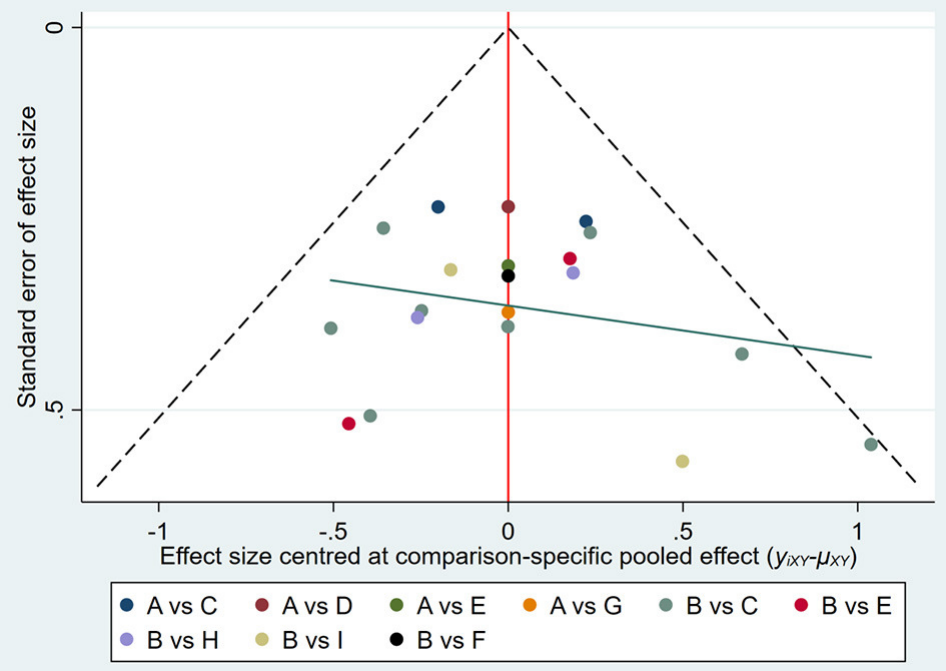
Funnel plot of publication bias of included studies. A, CT; B, sham; C, 20 min + 1 mA anodal; D, 20 min + 1.2 mA anodal; E, 20 min + 1.4 mA anodal; F, 20 min + 1.5 mA anodal; G, 20 min + 1.6 mA anodal; H, 20 min + 2 mA anodal; I, 30 min + 2 mA anodal.

**Figure 8:**
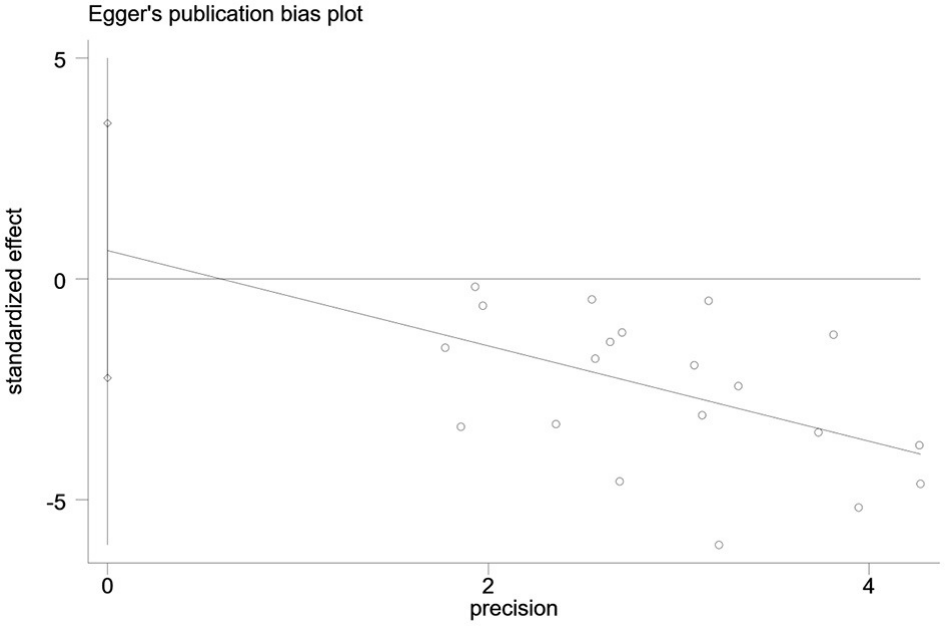
Egger's test of publication bias of included studies.

Publication bias in the outcome measures was analyzed. All studies were symmetrically distributed across the x = 0 line, which indicates that there was no small sample size effect or publication bias in the studies. Egger's test showed *P* = 0.646, supporting the absence of publication bias (Figures 7, 8).

## Discussion

Several regions of the brain are damaged after stroke, especially the motor and sensory cortices of the brainstem, thalamus, basal ganglia, and cerebellum, which have been implicated in the control of spontaneous and involuntary swallowing (49, 50). Previous studies showed that dysphagia is an independent risk factor for prolonged hospitalization and occurrence of complications during hospitalization (51, 52). Therefore, early diagnosis and treatment are critical for post-stroke dysphagia. There are numerous swallowing rehabilitation methods that promote patient recovery. Most traditional direct meta-analyses have compared anodal tDCS with CT or sham tDCS (53, 54), but there is currently no high-quality evidence showing the efficacy of different stimulation intensities and durations of anodal tCDS in the treatment of post-stroke dysphagia. In this study, we performed a network meta-analysis to indirectly compare the efficacy of different intervention protocols using common controls, and combined the direct and indirect evidence on different stimulation durations and intensities of anodal tCDS to obtain a probability ranking and hence the optimal regimen. The findings of study can provide insight to the optimal stimulation duration and intensity of anodal tDCS for promoting recovery of swallowing functions in stroke patients.

Our study included 20 RCTs involving 838 participants who were randomly assigned to receive different stimulation duration and intensities of anodal tDCS. Our results showed that 20 min of 1.4 mA anodal tDCS may be most beneficial for the recovery of swallowing functions in stroke patients. This finding demonstrates that the efficacy of anodal tDCS in post-stroke dysphagia is not improved by increasing stimulation duration and intensity, which is consistent with the lack of positive correlation between treatment efficacy and stimulation intensity in another meta-analysis (54). In addition, we should acknowledge that any current stimulation protocol has its own risk and may lead to problems under certain circumstances. There are still many issues that need to be addressed by extensive research and clinical practice. In general, the stimulation duration of tDCS ranges from 3 to 40 min (55) and a current intensity of 1–2 mA is considered to be safe in humans (56). Some of the studies in our analysis did not report any adverse reactions of tDCS in post-stroke dysphagia, this may be attributed to the small sample size. However, a large-cohort study has reported pain, fatigue and pruritus in healthy subjects and patients with other diseases who received tDCS (57). Similar adverse effects were also reported in another safety study of tDCS in stroke (58). Nonetheless, the safety of tDCS has been validated in numerous studies and the currently used stimulation parameters should be safe and reliable. All of the current intensities employed in the included studies were within 1–2 mA. Many studies compared different current intensities, but most of them investigated a stimulation duration of 20 min, except for 1 study that examined a stimulation duration of 30 min. It is possible that when the stimulation intensity is within the safe range, a change in the stimulation duration may impact the efficacy of tDCS in post-stroke dysphagia. However, further studies of stimulation duration and network meta-analyses of different stimulation durations and intensities will be warranted to identify the best anodal tDCS protocol.

Before our study, a previous meta-analysis of 15 RCTs found that tDCS could promote the recovery of swallowing function in post-stroke patients, and high-intensity stimulation might have a better effect (19). Conversely, another meta-analysis of 7 RCTs reported that low-intensity stimulation was more effective in improving dysphagia in patients with stroke (54). The two meta-analyses only explored the effects of stimulation intensity on the improvement of dysphagia but did not comprehensively consider the duration of stimulation. Meanwhile, both of them had a limited number of included studies. In contrast, our study had a larger sample size of updated RCT data, comprehensively analyzed the effects of intensity and duration of stimulation on dysphagia in patients with stroke, and elucidated the results using a consistency model. Furthermore, we performed a surface under the cumulative ranking (SUCRA) analysis for all eligible interventions, which showed that 20 min 1.4 mA transcranial direct current positive electrical stimulation therapy may be the most effective in improving dysphagia in patients with stroke.

There are several limitations in this study. First, the included RCTs performed swallowing exercises for both the treatment and control groups. The slight differences among the various exercises made them difficult to standardize among the studies. In addition, we did not examine the duration of swallowing exercise in the treatment, and control groups of each study. Since swallowing exercise is the current standard treatment, removing such treatment from stroke patients for the purpose of the study objective is unethical. These factors have impeded us from further assessing the impact of swallowing exercises on different stimulation intensities and durations of anodal tDCS. Second, the type of stroke varied among patients in the included RCTs. We cannot exclude the possible effect of different stroke type on the efficacy of different stimulation intensities and durations of anodal tDCS. Third, the instruments used in each study may differ. Only a few studies have reported the name and model of the instruments used, and variations among the instruments may lead to differences in treatment method and hence affect the study results. Fourth, only a limited number of studies provided long-term follow-up data for comparing the lasting effects of different stimulation intensities and durations of anodal tDCS. Fifth, we did not include other important outcomes, such as overall treatment efficacy, nutrition-related measures, and infection-related measures. These important outcomes were barely reported and thus we did not extract the relevant data. Finally, the methodological quality of RCT is generally low. In addition, since economic data was not reported, the cost of different tDCS instruments could not be evaluated. For future studies, we should (1) further validate the findings of this study by strictly following the RCT design, implementation and descriptions and ensuring the quality of the original study; (2) conduct RCT that directly compare tDCS protocols with different stimulation intensities and durations to make up for the drawbacks of indirect comparison; (3) use objective measures such as nutrition- and infection-related measures to make the evaluation of tDCS more objective; and 4) add economic assessment to obtain the most cost-effective current intensity for tDCS.

## Conclusion

In this network meta-analysis, we showed that different stimulation intensities of the intervention had different advantages in promoting the recovery of swallowing functions in stroke patients, but the difference in efficacy was not significant. Probability ranking revealed that 20 min of 1.4 mA tDCS may be the most beneficial protocol. However, due to the heterogeneity among studies, caution is required when interpreting these results. Clinicians should develop individualized treatment plans based on the patients' conditions and sensitivity to tDCS in order to bring out the optimal therapeutic effect of tDCS. Given that most included studies focused on a stimulation duration of 20 min, future studies can explore the impact of stimulation duration on the recovery of swallowing functions in stroke patients.

## Data availability statement

The original contributions presented in the study are included in the article/Supplementary material, further inquiries can be directed to the corresponding author.

## Author contributions

JX and XL contributed to conception and design of the study and organized the database. CZ and GN performed the statistical analysis. JX and XL wrote the first draft of the manuscript. CZ, GN, MW, and XJ wrote sections of the manuscript. All authors contributed to manuscript revision, read, and approved the submitted version.

## Supplementary material

Supplementary material 1 PRISMA_2020_checklist.

Supplementary material 2 The search strategy in all databases.
