# Supplementary material for: Dosage consideration for transcranial direct current stimulation in post-stroke dysphagia: A systematic review and network meta-analysis
Source: Front Neurol. 2023 Jan 24;14:1098831. doi: 10.3389/fneur.2023.1098831 (PMC9902951; doi:10.3389/fneur.2023.1098831)
Supplement: Supplementary material 2 — The search strategy in all databases. [file Data_Sheet_2.DOCX]

**Supplementary Material 2 |** The search strategy in all databases.

**Search strategy in PubMed**

| Search number | Query | Search Details | Results |
| --- | --- | --- | --- |
| 7 | (#1 OR #2) AND (#3 OR #4) AND (#5 OR #6) | ("stroke"[MeSH Terms] OR ("Acute Cerebrovascular Accident"[Title/Abstract] OR "Acute Cerebrovascular Accidents"[Title/Abstract] OR "acute cerebrovascular lesion"[Title/Abstract] OR "acute focal cerebral vasculopathy"[Title/Abstract] OR "Acute Stroke"[Title/Abstract] OR "Acute Strokes"[Title/Abstract] OR "apoplectic stroke"[Title/Abstract] OR "apoplexia"[Title/Abstract] OR "Apoplexy"[Title/Abstract] OR "brain attack"[Title/Abstract] OR "brain insult"[Title/Abstract] OR "Brain Vascular Accident"[Title/Abstract] OR "Brain Vascular Accidents"[Title/Abstract] OR "cerebral insult"[Title/Abstract] OR "Cerebral Strokes"[Title/Abstract] OR "cerebral vascular accident"[Title/Abstract] OR "cerebral vascular insufficiency"[Title/Abstract] OR "cerebro vascular accident"[Title/Abstract] OR "Cerebrovascular Accident"[Title/Abstract] OR "Cerebrovascular Accidents"[Title/Abstract] OR "Cerebrovascular Apoplexy"[Title/Abstract] OR "cerebrovascular failure"[Title/Abstract] OR "cerebrovascular injury"[Title/Abstract] OR "cerebrovascular insufficiency"[Title/Abstract] OR "cerebrovascular insult"[Title/Abstract] OR "Cerebrovascular Stroke"[Title/Abstract] OR "Cerebrovascular Strokes"[Title/Abstract] OR "cryptogenic stroke"[Title/Abstract] OR "CVA"[Title/Abstract] OR "CVAs"[Title/Abstract] OR "insultus cerebralis"[Title/Abstract] OR "ischemic seizure"[Title/Abstract] OR "stroke"[Title/Abstract] OR "Strokes"[Title/Abstract] OR "thrombotic stroke"[Title/Abstract])) AND ("deglutition disorders"[MeSH Terms] OR ("aphagopraxia"[Title/Abstract] OR "deglutition difficulty"[Title/Abstract] OR "Deglutition Disorder"[Title/Abstract] OR "deglutition disorders"[Title/Abstract] OR "difficult deglutition"[Title/Abstract] OR "difficulty in swallowing"[Title/Abstract] OR "difficulty swallowing"[Title/Abstract] OR "Dysphagia"[Title/Abstract] OR "dysphagias"[Title/Abstract] OR "Esophageal Dysphagia"[Title/Abstract] OR "Oropharyngeal Dysphagia"[Title/Abstract] OR "swallowing difficult"[Title/Abstract] OR "swallowing difficulty"[Title/Abstract] OR "Swallowing Disorders"[Title/Abstract])) AND ("transcranial direct current stimulation"[MeSH Terms] OR ("Anodal Stimulation tDCS"[Title/Abstract] OR "Anodal Stimulation Transcranial Direct Current Stimulation"[Title/Abstract] OR "Cathodal Stimulation tDCS"[Title/Abstract] OR "Repetitive Transcranial Electrical Stimulation"[Title/Abstract] OR "tDCS"[Title/Abstract] OR "Transcranial Alternating Current Stimulation"[Title/Abstract] OR "transcranial direct current stimulation"[Title/Abstract] OR "Transcranial Electrical Stimulation"[Title/Abstract] OR "Transcranial Electrical Stimulations"[Title/Abstract] OR "Transcranial Random Noise Stimulation"[Title/Abstract])) | 47 |
| 6 | "Anodal Stimulation tDCS"[Title/Abstract] OR "Anodal Stimulation tDCSs"[Title/Abstract] OR "Anodal Stimulation Transcranial Direct Current Stimulation"[Title/Abstract] OR "Cathodal Stimulation tDCS"[Title/Abstract] OR "Cathodal Stimulation tDCSs"[Title/Abstract] OR "Cathodal Stimulation Transcranial Direct Current Stimulation"[Title/Abstract] OR "Repetitive Transcranial Electrical Stimulation"[Title/Abstract] OR "tDCS"[Title/Abstract] OR "Transcranial Alternating Current Stimulation"[Title/Abstract] OR "transcranial direct current stimulation"[Title/Abstract] OR "Transcranial Electrical Stimulation"[Title/Abstract] OR "Transcranial Electrical Stimulations"[Title/Abstract] OR "Transcranial Random Noise Stimulation"[Title/Abstract] | "Anodal Stimulation tDCS"[Title/Abstract] OR "Anodal Stimulation Transcranial Direct Current Stimulation"[Title/Abstract] OR "Cathodal Stimulation tDCS"[Title/Abstract] OR "Repetitive Transcranial Electrical Stimulation"[Title/Abstract] OR "tDCS"[Title/Abstract] OR "Transcranial Alternating Current Stimulation"[Title/Abstract] OR "transcranial direct current stimulation"[Title/Abstract] OR "Transcranial Electrical Stimulation"[Title/Abstract] OR "Transcranial Electrical Stimulations"[Title/Abstract] OR "Transcranial Random Noise Stimulation"[Title/Abstract] | 7,726 |
| 5 | Transcranial Direct Current Stimulation[MeSH Terms] | "transcranial direct current stimulation"[MeSH Terms] | 4,269 |
| 4 | "aphagopraxia"[Title/Abstract] OR "deglutition difficulty"[Title/Abstract] OR "Deglutition Disorder"[Title/Abstract] OR "deglutition disorders"[Title/Abstract] OR "difficult deglutition"[Title/Abstract] OR "difficulty in swallowing"[Title/Abstract] OR "difficulty swallowing"[Title/Abstract] OR "Dysphagia"[Title/Abstract] OR "dysphagias"[Title/Abstract] OR "Esophageal Dysphagia"[Title/Abstract] OR "Oropharyngeal Dysphagia"[Title/Abstract] OR "swallowing difficult"[Title/Abstract] OR "swallowing difficultness"[Title/Abstract] OR "swallowing difficulty"[Title/Abstract] OR "Swallowing Disorders"[Title/Abstract] | "aphagopraxia"[Title/Abstract] OR "deglutition difficulty"[Title/Abstract] OR "Deglutition Disorder"[Title/Abstract] OR "deglutition disorders"[Title/Abstract] OR "difficult deglutition"[Title/Abstract] OR "difficulty in swallowing"[Title/Abstract] OR "difficulty swallowing"[Title/Abstract] OR "Dysphagia"[Title/Abstract] OR "dysphagias"[Title/Abstract] OR "Esophageal Dysphagia"[Title/Abstract] OR "Oropharyngeal Dysphagia"[Title/Abstract] OR "swallowing difficult"[Title/Abstract] OR "swallowing difficulty"[Title/Abstract] OR "Swallowing Disorders"[Title/Abstract] | 35,436 |
| 3 | Deglutition Disorders[MeSH Terms] | "deglutition disorders"[MeSH Terms] | 57,376 |
| 2 | "Acute Cerebrovascular Accident"[Title/Abstract] OR "Acute Cerebrovascular Accidents"[Title/Abstract] OR "acute cerebrovascular lesion"[Title/Abstract] OR "acute focal cerebral vasculopathy"[Title/Abstract] OR "Acute Stroke"[Title/Abstract] OR "Acute Strokes"[Title/Abstract] OR "apoplectic stroke"[Title/Abstract] OR "apoplexia"[Title/Abstract] OR "Apoplexy"[Title/Abstract] OR "brain accident"[Title/Abstract] OR "brain attack"[Title/Abstract] OR "brain blood flow disturbance"[Title/Abstract] OR "brain insult"[Title/Abstract] OR "brain insultus"[Title/Abstract] OR "Brain Vascular Accident"[Title/Abstract] OR "Brain Vascular Accidents"[Title/Abstract] OR "cerebral apoplexia"[Title/Abstract] OR "cerebral insult"[Title/Abstract] OR "Cerebral Strokes"[Title/Abstract] OR "cerebral vascular accident"[Title/Abstract] OR "cerebral vascular insufficiency"[Title/Abstract] OR "cerebro vascular accident"[Title/Abstract] OR "Cerebrovascular Accident"[Title/Abstract] OR "Cerebrovascular Accidents"[Title/Abstract] OR "Cerebrovascular Apoplexy"[Title/Abstract] OR "cerebrovascular arrest"[Title/Abstract] OR "cerebrovascular failure"[Title/Abstract] OR "cerebrovascular injury"[Title/Abstract] OR "cerebrovascular insufficiency"[Title/Abstract] OR "cerebrovascular insult"[Title/Abstract] OR "Cerebrovascular Stroke"[Title/Abstract] OR "Cerebrovascular Strokes"[Title/Abstract] OR "cerebrum vascular accident"[Title/Abstract] OR "cryptogenic stroke"[Title/Abstract] OR "CVA"[Title/Abstract] OR "CVAs "[Title/Abstract] OR "insultus cerebralis"[Title/Abstract] OR "ischaemic seizure"[Title/Abstract] OR "ischemic seizure"[Title/Abstract] OR "stroke"[Title/Abstract] OR "Strokes"[Title/Abstract] OR "thrombotic stroke"[Title/Abstract] | "Acute Cerebrovascular Accident"[Title/Abstract] OR "Acute Cerebrovascular Accidents"[Title/Abstract] OR "acute cerebrovascular lesion"[Title/Abstract] OR "acute focal cerebral vasculopathy"[Title/Abstract] OR "Acute Stroke"[Title/Abstract] OR "Acute Strokes"[Title/Abstract] OR "apoplectic stroke"[Title/Abstract] OR "apoplexia"[Title/Abstract] OR "Apoplexy"[Title/Abstract] OR "brain attack"[Title/Abstract] OR "brain insult"[Title/Abstract] OR "Brain Vascular Accident"[Title/Abstract] OR "Brain Vascular Accidents"[Title/Abstract] OR "cerebral insult"[Title/Abstract] OR "Cerebral Strokes"[Title/Abstract] OR "cerebral vascular accident"[Title/Abstract] OR "cerebral vascular insufficiency"[Title/Abstract] OR "cerebro vascular accident"[Title/Abstract] OR "Cerebrovascular Accident"[Title/Abstract] OR "Cerebrovascular Accidents"[Title/Abstract] OR "Cerebrovascular Apoplexy"[Title/Abstract] OR "cerebrovascular failure"[Title/Abstract] OR "cerebrovascular injury"[Title/Abstract] OR "cerebrovascular insufficiency"[Title/Abstract] OR "cerebrovascular insult"[Title/Abstract] OR "Cerebrovascular Stroke"[Title/Abstract] OR "Cerebrovascular Strokes"[Title/Abstract] OR "cryptogenic stroke"[Title/Abstract] OR "CVA"[Title/Abstract] OR "CVAs"[Title/Abstract] OR "insultus cerebralis"[Title/Abstract] OR "ischemic seizure"[Title/Abstract] OR "stroke"[Title/Abstract] OR "Strokes"[Title/Abstract] OR "thrombotic stroke"[Title/Abstract] | 307,513 |
| 1 | stroke[MeSH Terms] | "stroke"[MeSH Terms] | 160,389 |

**Search strategy in Embase**

| No. | Query | Results |
| --- | --- | --- |
| #7 | (#1 OR #2) AND (#3 OR #4) AND (#5 OR #6) | 79 |
| #6 | 'transcranial direct current stimulation'/exp | 9353 |
| #5 | 'anodal stimulation tdcs':ti,ab,kw OR 'anodal stimulation tdcss':ti,ab,kw OR 'anodal stimulation transcranial direct current stimulation':ti,ab,kw OR 'cathodal stimulation tdcs':ti,ab,kw OR 'cathodal stimulation tdcss':ti,ab,kw OR 'cathodal stimulation transcranial direct current stimulation':ti,ab,kw OR 'repetitive transcranial electrical stimulation':ti,ab,kw OR 'tdcs':ti,ab,kw OR 'transcranial alternating current stimulation':ti,ab,kw OR 'transcranial direct current stimulation':ti,ab,kw OR 'transcranial electrical stimulation':ti,ab,kw OR 'transcranial electrical stimulations':ti,ab,kw OR 'transcranial random noise stimulation':ti,ab,kw | 11052 |
| #4 | 'dysphagia'/exp | 88637 |
| #3 | 'aphagopraxia':ti,ab,kw OR 'deglutition difficulty':ti,ab,kw OR 'deglutition disorder':ti,ab,kw OR 'deglutition disorders':ti,ab,kw OR 'difficult deglutition':ti,ab,kw OR 'difficulty in swallowing':ti,ab,kw OR 'difficulty swallowing':ti,ab,kw OR 'dysphagia':ti,ab,kw OR 'dysphagias':ti,ab,kw OR 'esophageal dysphagia':ti,ab,kw OR 'oropharyngeal dysphagia':ti,ab,kw OR 'swallowing difficult':ti,ab,kw OR 'swallowing difficultness':ti,ab,kw OR 'swallowing difficulty':ti,ab,kw OR 'swallowing disorders':ti,ab,kw | 61511 |
| #2 | 'cerebrovascular accident'/exp | 385668 |
| #1 | 'acute cerebrovascular accident':ti,ab,kw OR 'acute cerebrovascular accidents':ti,ab,kw OR 'acute cerebrovascular lesion':ti,ab,kw OR 'acute focal cerebral vasculopathy':ti,ab,kw OR 'acute stroke':ti,ab,kw OR 'acute strokes':ti,ab,kw OR 'apoplectic stroke':ti,ab,kw OR 'apoplexia':ti,ab,kw OR 'apoplexy':ti,ab,kw OR 'brain accident':ti,ab,kw OR 'brain attack':ti,ab,kw OR 'brain blood flow disturbance':ti,ab,kw OR 'brain insult':ti,ab,kw OR 'brain insultus':ti,ab,kw OR 'brain vascular accident':ti,ab,kw OR 'brain vascular accidents':ti,ab,kw OR 'cerebral apoplexia':ti,ab,kw OR 'cerebral insult':ti,ab,kw OR 'cerebral strokes':ti,ab,kw OR 'cerebral vascular accident':ti,ab,kw OR 'cerebral vascular insufficiency':ti,ab,kw OR 'cerebro vascular accident':ti,ab,kw OR 'cerebrovascular accident':ti,ab,kw OR 'cerebrovascular accidents':ti,ab,kw OR 'cerebrovascular apoplexy':ti,ab,kw OR 'cerebrovascular arrest':ti,ab,kw OR 'cerebrovascular failure':ti,ab,kw OR 'cerebrovascular injury':ti,ab,kw OR 'cerebrovascular insufficiency':ti,ab,kw OR 'cerebrovascular insult':ti,ab,kw OR 'cerebrovascular stroke':ti,ab,kw OR 'cerebrovascular strokes':ti,ab,kw OR 'cerebrum vascular accident':ti,ab,kw OR 'cryptogenic stroke':ti,ab,kw OR 'cva':ti,ab,kw OR 'cvas':ti,ab,kw OR 'insultus cerebralis':ti,ab,kw OR 'ischaemic seizure':ti,ab,kw OR 'ischemic seizure':ti,ab,kw OR 'stroke':ti,ab,kw OR 'strokes':ti,ab,kw OR 'thrombotic stroke':ti,ab,kw | 492550 |

**Search strategy in The Cochrane Library**

| ID | Search | Results |
| --- | --- | --- |
| #1 | ('Acute Cerebrovascular Accident' OR 'Acute Cerebrovascular Accidents' OR 'acute cerebrovascular lesion' OR 'acute focal cerebral vasculopathy' OR 'Acute Stroke' OR 'Acute Strokes' OR 'apoplectic stroke' OR 'apoplexia' OR 'Apoplexy' OR 'brain accident' OR 'brain attack' OR 'brain blood flow disturbance' OR 'brain insult' OR 'brain insultus' OR 'Brain Vascular Accident' OR 'Brain Vascular Accidents' OR 'cerebral apoplexia' OR 'cerebral insult' OR 'Cerebral Strokes' OR 'cerebral vascular accident' OR 'cerebral vascular insufficiency' OR 'cerebro vascular accident' OR 'Cerebrovascular Accident' OR 'Cerebrovascular Accidents' OR 'Cerebrovascular Apoplexy' OR 'cerebrovascular arrest' OR 'cerebrovascular failure' OR 'cerebrovascular injury' OR 'cerebrovascular insufficiency' OR 'cerebrovascular insult' OR 'Cerebrovascular Stroke' OR 'Cerebrovascular Strokes' OR 'cerebrum vascular accident' OR 'cryptogenic stroke' OR 'CVA' OR 'CVAs ' OR 'insultus cerebralis' OR 'ischaemic seizure' OR 'ischemic seizure' OR 'stroke' OR 'Strokes' OR 'thrombotic stroke'):ti,ab,kw | 67466 |
| #2 | MeSH descriptor: [Stroke] explode all trees | 11470 |
| #3 | ('aphagopraxia' OR 'deglutition difficulty' OR 'Deglutition Disorder' OR 'deglutition disorders' OR 'difficult deglutition' OR 'difficulty in swallowing' OR 'difficulty swallowing' OR 'Dysphagia' OR 'dysphagias' OR 'Esophageal Dysphagia' OR 'Oropharyngeal Dysphagia' OR 'swallowing difficult' OR 'swallowing difficultness' OR 'swallowing difficulty' OR 'Swallowing Disorders'):ti,ab,kw | 5560 |
| #4 | MeSH descriptor: [Deglutition Disorders] explode all trees | 3114 |
| #5 | ('Anodal Stimulation tDCS' OR 'Anodal Stimulation tDCSs' OR 'Anodal Stimulation Transcranial Direct Current Stimulation' OR 'Cathodal Stimulation tDCS' OR 'Cathodal Stimulation tDCSs' OR 'Cathodal Stimulation Transcranial Direct Current Stimulation' OR 'Repetitive Transcranial Electrical Stimulation' OR 'tDCS' OR 'Transcranial Alternating Current Stimulation' OR 'transcranial direct current stimulation' OR 'Transcranial Electrical Stimulation' OR 'Transcranial Electrical Stimulations' OR 'Transcranial Random Noise Stimulation'):ti,ab,kw | 5705 |
| #6 | MeSH descriptor: [Transcranial Direct Current Stimulation] explode all trees | 1014 |
| #7 | (#1 OR #2) AND (#3 OR #4) AND (#5 OR #6) | 52 |

**Search strategy in Web of Science**

| ID | Search | Results |
| --- | --- | --- |
| #4 | #1 AND #2 AND #3 | 70 |
| #3 | "Stroke" OR "Acute Cerebrovascular Accident" OR "Acute Cerebrovascular Accidents" OR "acute cerebrovascular lesion" OR "acute focal cerebral vasculopathy" OR "Acute Stroke" OR "Acute Strokes" OR "apoplectic stroke" OR "apoplexia" OR "Apoplexy" OR "brain accident" OR "brain attack" OR "brain blood flow disturbance" OR "brain insult" OR "brain insultus" OR "Brain Vascular Accident" OR "Brain Vascular Accidents" OR "cerebral apoplexia" OR "cerebral insult" OR "Cerebral Strokes"OR "cerebral vascular accident" OR "cerebral vascular insufficiency" OR "cerebro vascular accident" OR "Cerebrovascular Accident" OR "Cerebrovascular Accidents" OR "Cerebrovascular Apoplexy" OR "cerebrovascular arrest" OR "cerebrovascular failure" OR "cerebrovascular injury" OR "cerebrovascular insufficiency" OR "cerebrovascular insult" OR "Cerebrovascular Stroke" OR "Cerebrovascular Strokes" OR "cerebrum vascular accident" OR "cryptogenic stroke" OR "CVA" OR "CVAs " OR "insultus cerebralis" OR "ischaemic seizure" OR "ischemic seizure" OR "Strokes" OR "thrombotic stroke"(Topic) | 878108 |
| #2 | "Deglutition Disorders" OR "aphagopraxia" OR "deglutition difficulty" OR "Deglutition Disorder" OR "deglutition disorders" OR "difficult deglutition" OR "difficulty in swallowing" OR "difficulty swallowing" OR "Dysphagia" OR "dysphagias" OR "Esophageal Dysphagia" OR "Oropharyngeal Dysphagia" OR "swallowing difficult" OR "swallowing difficultness"OR "swallowing difficulty" OR "Swallowing Disorders"(Topic) | 66520 |
| #1 | "Transcranial Direct Current Stimulation" OR "Anodal Stimulation tDCS" OR "Anodal Stimulation tDCSs" OR "Anodal Stimulation Transcranial Direct Current Stimulation" OR "Cathodal Stimulation tDCS" OR "Cathodal Stimulation tDCSs" OR "Cathodal Stimulation Transcranial Direct Current Stimulation" OR "Repetitive Transcranial Electrical Stimulation" OR "tDCS" OR "Transcranial Alternating Current Stimulation" OR "transcranial direct current stimulation" OR "Transcranial Electrical Stimulation" OR "Transcranial Electrical Stimulations" OR "Transcranial Random Noise Stimulation"(Topic) | 13754 |

**Search strategy in CNKI (In Chinese)**

(SU='Transcranial Direct Current Stimulation' OR SU=' Direct Current Stimulation') AND (SU=' Deglutition Disorders 'OR SU=' Dysphagia ') AND (SU='Stroke' OR SU=' Cerebrovascular Accident 'OR SU=' cerebral infarction' OR SU=' cerebral hemorrhage 'OR SU=' cerebrum vascular accident 'OR SU=' apoplexia' OR SU=' cerebral stroke ')

Scope of resources: general database; Synonym extension; Time range: starting year of inclusion to 2022-06-22;

Classification of literature: Medical and health technology

**Search strategy in SinoMed (In Chinese)**

Title or key words or subject term: (" Transcranial Direct Current Stimulation "OR" Direct Current Stimulation ") and (" Deglutition Disorders "OR" Dysphagia ") and (" Stroke "OR" Cerebrovascular Accident "OR" cerebral infarction "OR" cerebral hemorrhage "OR" cerebrum vascular accident "OR" apoplexia "OR" cerebral stroke ")

Qualification: Subject word extension, Chinese, Medicine, Time range: starting year of inclusion to 2022-06-22;

**Search strategy in Wanfang (In Chinese)**

Title or key words: (" Transcranial Direct Current Stimulation "or" Direct Current Stimulation ") and (" Deglutition Disorders "or" Dysphagia ") and (" Stroke "or" Cerebrovascular Accident "or" cerebral infarction "or" cerebral hemorrhage "or" cerebrum vascular accident "or" apoplexia "or" cerebral stroke ")

Qualification: Subject word extension, Chinese, Medicine, Time range: starting year of inclusion to 2022-06-22;

**Search strategy in WeiPu (In Chinese)**

Title or key words: (" Transcranial Direct Current Stimulation "OR" Direct Current Stimulation ") and (" Deglutition Disorders "OR" Dysphagia ") and (" Stroke "OR" Cerebrovascular Accident "OR" cerebral infarction "OR" cerebral hemorrhage "OR" cerebrum vascular accident "OR" apoplexia "OR" cerebral stroke ")

Subject limitation: Medical and health, Time range: starting year of inclusion to 2022-06-22.
